# Analytical Techniques for Supporting Hospital Case Mix Planning Encompassing Forced Adjustments, Comparisons, and Scoring

**DOI:** 10.3390/healthcare13010047

**Published:** 2024-12-30

**Authors:** Robert L. Burdett, Paul Corry, David Cook, Prasad Yarlagadda

**Affiliations:** 1 School of Mathematical Sciences, Queensland University of Technology, 2 George Street, P.O. Box 2434, Brisbane, QLD 4000, Australia; p.corry@qut.edu.au; 2 Princess Alexandra Hospital, 2 Ipswich Rd., Woolloongabba, Brisbane, QLD 4102, Australia; david.cook@health.qld.gov.au; 3 School of Engineering, University of Southern Queensland, Springfield, QLD 4300, Australia; y.prasad@usq.edu.au

**Keywords:** hospital case mix planning, multi-criteria decision-making, decision support tool, OR in health services

## Abstract

**Background/Objectives**: This article presents analytical techniques and a decision support tool to aid in hospital capacity assessment and case mix planning (CMP). To date, no similar techniques have been provided in the literature. **Methods**: Initially, an optimization model is proposed to analyze the impact of making a specific change to an existing case mix, identifying how patient types should be adjusted proportionately to varying levels of hospital resource availability. Subsequently, multi-objective decision-making techniques are introduced to compare and critique competing case mix solutions. **Results**: The proposed techniques are embedded seamlessly within an Excel Visual Basic for Applications (VBA) personal decision support tool (PDST), for performing informative quantitative assessments of hospital capacity. The PDST reports informative metrics of difference and reports the impact of case mix modifications on the other types of patients present. **Conclusions**: The techniques developed in this article provide a bridge between theory and practice that is currently missing and provides further situational awareness around hospital capacity.

## 1. Introduction

### 1.1. Background

This article explores the development of techniques that support case mix planning in hospitals. Case mix planning involves identifying a patient cohort (i.e., case mix) with a specific set of characteristics deemed desirable or optimal. Determining the ideal composition and number of patients to treat is a complex and nuanced task (Hof et al. [1]). There are many different alternative case mixes that can be selected. Some case mixes are favorable for some patient groupings (a.k.a., patient types) and unfavorable for others (Burdett et al. [2]). The term “ideal” is quite subjective and can vary in meaning, especially in a practical setting. A case mix may be sought that prioritizes equity, particularly in the allocation and utilization of hospital resources. A case mix may also be sought that is most cost-effective or financially viable to treat. From a utilization and output-oriented perspective, a maximal cohort may be sought—one that leads to the highest number of patients treated over time. This cohort effectively saturates the hospital’s resources and serves as a measure of the hospital’s capacity (Burdett et al. [2]). Identifying a case mix that meets or exceeds specified demands or targets is also of significant interest.

Case mix planning is challenging, further complicated by a lack of precise information, an abundance of unrefined empirical data, and the presence of stochastic parameters (Burdett et al. [3]). The presence of stochastic treatment durations and lengths of stay makes it difficult to accurately determine the exact utilization of each hospital resource over a specified period.

The categorization of patient types is a crucial component of CMP, but the approach is rarely straightforward. It is often ad-hoc and politically sensitive. The categorization of patient types can be politically sensitive because it often involves decisions about resource allocation, funding, and prioritization of care. These decisions can have significant impacts on different groups of patients, healthcare providers, and stakeholders, leading to debates about fairness, equity, and access to care. For example, prioritizing certain patient groups over others can create tensions among different departments, healthcare professionals, or patient advocacy groups, who may have competing interests or concerns about how resources are distributed. Additionally, political or policy considerations may influence how patient categories are defined and which groups are emphasized, further contributing to the sensitivity of the process.

There are numerous types of illnesses and a wide range of medical and surgical treatments. Categorizing patients into finite groups with shared characteristics is inherently subjective, and any classification can potentially skew the results. Patients may be grouped by specialty, diagnostic resource group (DRG), International Classification of Diseases (ICD), similar resource utilization, treatment duration, or other methods, each of which may influence outcomes in different ways (Andrews et al. [4]). In CMP there is a decision to include surgical, medical, or acute patients, and to approach the hospital either holistically or in a more fragmented manner. It is also important to note that CMP can refer to the allocation of available operating theatre time among different surgeons, surgical specialties, and types of surgical patients.

### 1.2. Past Research and Current State of the Art

Strategic, tactical, and operational decision-making challenges are abundant in healthcare, and substantial research has been conducted of late (Humphreys et al. [5]). Operational problems like scheduling are popular. Some noteworthy contributions include Chen et al. [6], Chen et al. [7], Spratt and Kozan [8], Liu et al. [9], Hawkinson et al. [10] and Zhou et al. [11]. Predicting patient flows is another vital task. It provides information that can be used in case mix planning and capacity assessment. Resta et al. [12] developed artificial neural networks to classify, cluster, and predict patient flows. They used their self-organizing map to gain a deeper understanding of patient flows in an emergency department in Italy.

Providing advanced predictive analytics is an important goal in the healthcare industry (Kreuger [13]), and the development of planning software that is user-friendly, intuitive, and extensible is desirable. Although Krueger [13] highlights the emerging use of operational tools and scientific methods in healthcare, it is important to recognize that the adoption and development of advanced hospital planning tools and software have been limited thus far (Burdett et al. [14]). Our current research has identified the following reasons limiting the adoption and development of advanced hospital planning tools and software:

1. Complexity and Customization: Healthcare systems are highly complex and vary widely across different hospitals, regions, and countries. Developing tools that are both sophisticated enough to address these complexities and flexible enough to be adapted to diverse settings is challenging. This often leads to slower adoption as hospitals must tailor solutions to their unique needs.
2. High Costs: Developing and implementing advanced planning tools can be expensive. Hospitals, particularly those with limited budgets, may prioritize other areas of investment, such as staffing or medical equipment, over advanced software solutions.
3. Resistance to Change: Healthcare organizations often have entrenched practices and workflows. There can be resistance from staff and management to adopt new technologies, particularly if these tools require significant changes to existing processes or a learning curve.
4. Data Availability and Integration: Effective hospital planning tools require access to high-quality, comprehensive data, including patient outcomes, resource utilization, and financial metrics. In many cases, data is siloed or inconsistent, making it difficult to integrate and use effectively in planning tools.
5. Regulatory and Privacy Concerns: Healthcare data is sensitive and subject to strict regulations (such as HIPAA in the U.S.). These regulations can create barriers to the development and deployment of tools that require large data sets or cross-organizational data sharing.
6. Lack of Skilled Personnel: Developing and maintaining advanced planning tools requires specialized knowledge in both healthcare and data science. There may be a shortage of professionals with the expertise necessary to design, implement, and manage these systems within hospitals.
7. Uncertainty of Return on Investment (ROI): Many healthcare administrators are cautious about investing in advanced planning tools without clear evidence of ROI. The potential benefits of such tools, such as improved efficiency or better resource allocation, may be difficult to quantify, leading to hesitation in adopting them.

These factors combined contribute to the slow pace of adoption and development in this area. Trust in the team responsible for building a tool has also been highlighted as crucial to its success (Pagel et al. [15]).

Regarding the main topic of this article, case mix planning is a relatively small and niche field, but it has gained increasing importance over the past decade, with the development of a variety of innovative quantitative methods. Those methods predominantly focus on identifying an optimal case mix, while accounting for resource availability constraints. They typically integrate key hospital parameters along with other geographic factors, such as hospital layout and configuration. Mixed integer programming has been the basis of previous methods, and both deterministic and stochastic parameters have been incorporated. Some of the decision models have included multiple criteria. The articles by Andrews et al. [4], Burdett et al. [2,3,14,16,17,18], Freeman et al. [19], McRae et al. [20], McRae and Brunner [21], and Mahmoudzadeh et al. [22] are representative of the current state of the art and should be consulted for further details. Burdett et al. [16] is particularly noteworthy for clarifying how CMP can be applied within a single hospital or to a region of hospitals. According to Bruggemann et al. [23], however, there is a lack of methodological support for health care professionals in their decision-making, encompassing the provision and allocation of finite hospital resources. Our review of the literature supports this conclusion. We have also observed that CMP across multiple hospitals has yet to be comprehensively addressed, and stochastic assessments are also still relatively new (Burdett et al. [3], Mahmoudzadeh et al. [22]). The development of appropriate decision support tools is severely lacking, and only the recent work by Burdett et al. [14] considers how hospital planners, executives, and laypersons could perform CMP. For that reason, this article considers how to embed some new methods into software for practical application.

This article identifies the potential for capacity-planning research in nursing and other community care facilities as an emerging area of focus. Frail and elderly patients are highly vulnerable and are often a cause of bed blocking within hospitals due to the lack of or unsuitable community care resources available (Williams et al. [24]). It is therefore important for research to focus on hospitals and how they feed into community care services.

Multi-criteria decision-making (MCDM) has increased greatly in healthcare in recent years (Chalgham et al. [25]). Chalgham et al. [25] proposed MCDM methods to improve in-patient flows from an emergency department. Malik et al. [26] formulated and solved a bi-objective aggregate capacity planning problem for operating theatres. They considered the minimization of elective patients waiting for treatment and minimization of healthcare expenditure. Both of the aforesaid articles, however, did not consider an entire hospital and the effect on downstream resources, like beds. Emergency management of health care facilities in the event of various disasters and crises is also an active area for which this articles’ approach is applicable. High-level emergencies have serious consequences on hospital activities (Chen, et al. [6]). Chen et al. [6] considered hospital evacuation planning and developed a simulation model to analyse the process.

### 1.3. Research Agenda

The need for additional techniques to support hospital CMP has been identified in past research and development activities. Hence, in this article we have proposed some additional mathematical techniques to fill that gap. First, we consider how a specific change to an existing patient case mix impacts the resource availability for other patient types. Specifically, we would like to know which patient types are affected, and by how much are they affected. Ultimately, we query whether more, or less patients can be treated. We think this functionality is critical because it allows us to assess how adjustments to the patient cohort can influence hospital operations, resource utilization, and overall patient care. By quantifying the effects on different patient types, we can better determine whether the hospital’s capacity can be expanded to treat more patients, or if adjustments are needed to treat fewer patients, while still maintaining or improving care quality. This analysis will help optimize resource allocation and inform strategic decisions on patient prioritization to achieve both operational efficiency and high-quality outcomes.

There are many qualitative factors, and some slight adjustment of the mix would typically be necessary to satisfy stakeholders. To identify the effect, we propose a mathematical programming model. The capability to analyse changes of this nature and to immediately see the effect is expected to improve situational awareness for hospital executives, managers, and planners who are weighing up the pros and cons of treating more or less patients of a given type. This type of enquiry in theory facilitates a broader sensitivity analysis that can provide significant insights into how a hospital can be operated, and who they can treat. Previously, the goal of CMP was just to identify a single favourable case mix solution, or to provide a set of non-dominated case mix solutions, with no understanding of the difference between them.

Second, we consider how to compare different patient case mixes to better facilitate CMP within a multi-criterion setting. In a multi-criterion setting, it is necessary to generate a Pareto frontier, which is a set of alternatively optimal solutions. How to choose one solution over another is very unclear. We would like to decide rationally, and to have a consistent framework to substantiate any choice. To this end, analytical aids are needed.

The techniques developed in this article motivate the development of a decision support tool for hospital managers and planners to use. Without such a tool, it is hard to imagine that the techniques advocated in this article, or many others, could be applied in a practical setting. It seems unlikely that any hospital or health care provider would have the time and resources to create a tool that does what this paper suggests, so it falls to us to prove the concept. We test how the new techniques can be embedded within a personal decision support tool to perform the described CMP analyses.

The structure of this article is as follows. In Section 2, the details of the analytical methods and framework is provided, commencing with an outline of key technical details. The specification, capabilities, and graphical user interfaces are then presented in Section 3. Design strategies employed during development are also provided, and examples of how the GUI are used to perform various assessments is shown. A covid inspired case study is presented in Section 4. Conclusions and final remarks are given in Section 5. Broader issues including extensions to the software are also discussed.

## 2. Methodology

In this section, several methodologies are introduced for CMP. The first pertains to case mix alterations and revisions and the quantification of impact. The second pertains to case mix comparisons and scoring. Before those are provided, background details and notations relevant to CMP are provided.

### 2.1. Background Details and Notations

To perform case mix planning, we require two things: First, information about the hospital infrastructure that is used to treat patients, and second, information regarding the patients to be treated. Operating rooms and wards have a significant impact on hospital outputs and constitute the main treatment areas (a.k.a., infrastructure). These are hereby denoted by set I. The number of treatment spaces (a.k.a., beds), within each i∈I is denoted $b_{i},$ and the time availability to treat patients is $T_{i}$ hours. The number of treatment spaces naturally affects $T_{i}$. Typically, $T_{i}=W\times h_{i},$ where W is the total number of weeks and $h_{i}$ is the hours operational per week. If the infrastructure is a ward, then $T_{i}=W\times h_{i}\times b_{i}$.

It is assumed that there is a clearly defined set of patient groups (a.k.a., types) that are treated. For each patient type g∈G, a set of patient subtypes, $P_{g}$, is defined. For each $g\in G,p\in P_{g}$ there is a patient pathway or profile, denoted $A_{g,p}$. Each patient pathway is a collection of activities. For each $a\in A_{g,p}$ there is a planned (i.e., expected) treatment time of $t_{a}$ hours and a set of candidate locations $I_{a}$ where $I_{a}\subset I$. We also define the set of activities as A and those permitted at location i as $A_{i}=\left\{a\in A|i\in I_{a}\right\}$.

The flow (a.k.a., number of) of patients of type g and the flow of patients of sub-type p are an output of CMP. These constitute the case mix and sub mix of the hospital. They are respectively denoted $n_{g}^{1}$ and $n_{g,p}^{2}$. The total flow of patients treated is ℕ. The following hierarchical relationships are worth noting: $\mathbb{N}=\underset{g}{\sum }n_{g}^{1}\;\forall g\in G$ and $n_{g}^{1}=\underset{p\in P_{g}}{\sum }\left(n_{g,p}^{2}\right)\;\forall g\in G,\forall p\in P_{g}$. The number of activities a∈A performed at i∈I is denoted $\beta_{a,i}.$ Naturally, $\beta_{a,i}=0\;\forall i\in I\backslash I_{a}$, as activities cannot be performed outside of their candidate locations. The decision captured by $\beta_{a,i}$ is called the allocation. It is inherently linked to the decisions $n_{g}^{1}$ and $n_{g,p}^{2}$.

The modelling from [2,3,14,16,17,18] has been used as the basis of this article’s new quantitative techniques. The model shown in Appendix A determines the maximum flow of patients over a specified period, subject to limited time availabilities of operating theatres, wards, and beds, and required patient type proportions.

### 2.2. Impact of Case Mix Alterations

In this section, an approach to perform a case mix alteration assessment (CMAA) is provided. For a given case mix $n^{1}$, the effect of a change in the number of patients of a single type $g^{*}$ is worth understanding. If patient type $g^{*}$ is increased and “anchored”, then it may be necessary to decrease several other patient types, as less capacity is available for them. If patient type $g^{*}$ is decreased and anchored, then the opposite occurs, i.e., more capacity is available for the others. The following algorithm is proposed to identify the impact of an alteration (Algorithm 1):
**Algorithm 1.** Impact AssessmentStep 1. Select a case mix for analysis, hereby $n^{1}$, and designate targets ${\hat{n}}_{g}^{1}=n_{g}^{1}$.Step 2. Select a patient type $g^{*}$, and force a change of δ units, where $0\leq n_{g^{*}}^{1}+\delta \leq {\bar{n}}_{g^{*}}^{1}$ and δ≠0
Step 3. If δ>0 then differences in the case mix should be minimized, i.e., the 2-norm ${\left\|n^{1}-{\hat{n}}^{1}\right\|}_{2}$ should be minimized, or else it should be maximized, where ${\left\|n^{1}-{\hat{n}}^{1}\right\|}_{2}={\left(\underset{g\in G}{\sum }{\left(\gamma_{g}\right)}^{2}\right)}^{0.5}$ and $\gamma_{g}={\hat{n}}_{g}^{1}-n_{g}^{1}$ or $\gamma_{g}=\left({\hat{n}}_{g}^{1}-n_{g}^{1}\right)/{\bar{n}}_{g}^{1}$.Step 4. Solve the optimization model below, and report the case mix $n^{1}$ and the differences $\gamma_{g}$.   Minimize $Z=\Delta \times \underset{g\in G\backslash \left\{g^{*}\right\}}{\sum }{\left(\gamma_{g}\right)}^{2}$ where Δ=sign(δ)=δ/|δ|
(1)   Subject to:   $n_{g^{*}}^{1}={\hat{n}}_{g^{*}}^{1}+\delta$[Anchored value](2)   
$\gamma_{g^{*}}=0$


   
$\gamma_{g}=\Delta \times \left({\hat{n}}_{g}^{1}-n_{g}^{1}\right)/{\bar{n}}_{g}^{1}$
$\forall g\in G\backslash \left\{g^{*}\right\}$[Scaled difference](3)   
$\Delta \times n_{g}^{1}\leq \Delta \times {\hat{n}}_{g}^{1}$$\forall g\in G\backslash \left\{g^{*}\right\}$
[Forced increase or decrease]

(4)   
$\gamma_{g}\geq 0$∀g∈G
[Positive scaled difference](5)   Constraints (A2)–(A7) from Appendix A

The model listed at Step 4 is hereby denoted “CMAA-SSQ” as it has a sum of squares objective. The main decision variable of the model is denoted $\gamma_{g}$. It is used to record the actual change (or relative change) in the number of patients of type *g* treated. Notably, scaling is introduced in constraint (3) to manage the different orders of magnitude that may occur. As such, differences can be compared more objectively. Without scaling, some types will have more or less increase (decrease). Equation (3) also ensures that $\gamma_{g}$ takes positive values in both situations. For instance, $\forall g\in G\backslash \left\{g^{*}\right\}$: $\gamma_{g}=\left({\hat{n}}_{g}^{1}-n_{g}^{1}\right)/{\bar{n}}_{g}^{1}$ if δ>0 and $\gamma_{g}=\left(n_{g}^{1}-{\hat{n}}_{g}^{1}\right)/{\bar{n}}_{g}^{1}\;\mathrm{if}\;\delta <0$. Constraint (4) ensures that a patient type is not increased when it should be decreased, or vice versa. For example, if δ<0, then there must be an increase, i.e., $n_{g}^{1}\geq {\hat{n}}_{g}^{1}$. If δ>0, then there must be a decrease, i.e., $n_{g}^{1}\leq {\hat{n}}_{g}^{1}$.

As the sum of squares objective is a quadratic function, the model can be solved using Quadratic Programming (QP) techniques. However, when δ<0, we find the model to be non-convex (i.e., the hessian is not positive semi-definite), and QP is not suitable. Separable Programming (SP) techniques, however, may be applied. This requires the objective to be expanded as shown in (6) and the term ${\left(n_{g}^{1}/{\bar{n}}_{g}^{1}\right)}^{2}$ to be approximated by a piecewise linear function $\zeta_{g}=\mathrm{PWL}\left(n_{g}^{1}/{\bar{n}}_{g}^{1},f,b,\sigma \right)$ where $b_{0}=0$, $b_{i}=\frac{i}{I+1}\;i=1,\ldots ,I+1$, $\sigma_{i}=\frac{f(b_{i})-f\left(b_{i-1}\right)}{b_{i}-b_{i-1}}\;i=1,\ldots ,I+1$, and $f\left(b\right)=b^{2}$.

(6) $$\sum_{g|g\neq g^{*}}{\left(\gamma_{g}\right)}^{2}=\sum_{g\in G\left\{g^{*}\right\}}{\left(n_{g}^{1}/{\bar{n}}_{g}^{1}\right)}^{2}-2{\hat{n}}_{g}^{1}\left(n_{g}^{1}/{\bar{n}}_{g}^{1}\right)+{\left({\hat{n}}_{g}^{1}/{\bar{n}}_{g}^{1}\right)}^{2}$$

In total, there is |G|−1 piecewise linear functions. The term $n_{g}^{1}/{\bar{n}}_{g}^{1}$ lies in the range [0,1], and because of this, so does the term ${\left(n_{g}^{1}/{\bar{n}}_{g}^{1}\right)}^{2}$. The squared term is well-approximated by a piecewise linear function over that interval and requires a relatively small number of intervals and breakpoints. There are other alternatives to the sum of squares approach. Enforcing a more equitable change across all types is an option. The following linear objective may instead be considered:

(7) $$\mathrm{Minimize}\;Z=\sum_{g\in G}\left(\gamma_{g}\right)$$

This variation is hereby denoted “CMAA-LIN”. This approach still favors some types and not others and is not necessarily equitable, but it may be reasonable in some circumstances. Another alternative is to explicitly force an equitable increase or decrease. The following objective and constraint can be imposed to achieve this:

(8) $$\mathrm{Minimize}\;Z=\Delta \times \lambda \mathrm{where}\;\gamma_{g}=\lambda \;\forall g\in G\backslash \left\{g^{*}\right\}$$

The scaling previously discussed is vital for using the objective function in (8). Furthermore, the equality $\gamma_{g}=\lambda$ is strict, and that might be an issue sometimes. For instance, a particular patient type may not be able to be increased or decreased at all, as required resources may already be fully utilized, or the lower or upper bounds reached. In response, we can instead enforce $\gamma_{g}\leq \lambda$, and add the following condition:

(9) $$n_{g}^{1}\geq {\hat{n}}_{g}^{1}-\Delta \times \lambda {\bar{n}}_{g}^{1}\;\forall g\in G\backslash \left\{g^{*}\right\}$$

This variation is hereby denoted “CMAA -EQ”. The right-hand side of (9) must be positive. As such, a bound for λ can be computed, i.e., $\lambda \leq \left({\hat{n}}_{g}^{1}/{\bar{n}}_{g}^{1}\right)/\Delta \;\forall g\in G$.

**Additional Remarks:** The model may identify no changes are necessary after an alteration. However, a different allocation of resources may be necessary to achieve the alteration. Hence, the model outputs are still valuable. Also worth noting, the model may not be feasible in some circumstances, and that is informative of situations where an equitable alteration is not possible. An inequitable alteration, however, may be possible, but that requires a more sophisticated multicriteria approach and some additional direction and guidance about how to regulate competition between the different patient types.

Several examples demonstrating the application of the above methodology is now presented:

**Example 1.**  *In this section, a scenario is provided to demonstrate case mix alteration assessment. In this scenario, we consider a small facility with five intensive care beds, 10 operating theatres, five recovery wards with (2, 5, 10, 14, 3) beds, respectively, and five patient types whose details are provided in Table 1. To start the process, the case mix* $n^{1}=$ *(5.68, 48.82, 20.43, 10.22, 28.38) with a flow of 113.53 patients per week was arbitrarily selected. The case mix proportions are respectively (0.05, 0.43, 0.18, 0.09, 0.25).* *Table 2, Table 3, Table 4 and Table 5, describe the effect of various alterations and the solution of the variant objectives. The results are rounded to two decimal places. Changes to specific patient types are shown in column two, and the change to all other patient types is shown in column four. In column seven, the total corresponding change in the other patient types is shown, i.e., the impact is the sum of the alterations in column 4, excluding those attributed to the selected patient type. Depending on the method, we also show*λ*and the objective function value*Z*. For brevity, only a small number of revisions are entertained. The considered number is, however, sufficient to gain a basic understanding of the range of impacts. A more thorough and methodical analysis could be performed if there is need. We have, however, considered what would happen if a patient type was eliminated completely or increased to its upper bound. Note that “ic”, “sur”, “postop” refer to intensive care, surgery and postoperative care respectively.*

From the CMAA-EQ results in Table 2, it is worth noting a few things. It is possible that some requested changes reduce all other patient types to zero. Also, if a patient type is increased too greatly, then some patient types would need to be reduced below zero to achieve a uniform equitable decrease. This is not permitted, however, and the model is not solvable in those circumstances. For example, when patient type T5 is increased to a level greater than 57.6, then it is impossible to achieve a uniform decrease. To obtain a solution, it is necessary to include constraint (9). Hence, patient type T4 is decreased as much as possible, while the others are decreased equitably. The gamma value for patient type T4 is 0.097289 for all changes in patient type T5 above 57.6. When the increase is 57.9, then lambda is 0.0982 and the value of $n_{4}^{1}=0.0982\times 105.047=10.3156$, which is greater than the original value of 10.22.

In Table 4 and Table 5, the original CMAA-SSQ approach is demonstrated. In Table 4 the results were obtained via quadratic programming, however, in Table 5 separable programming. The number of breakpoints used to approximate the squared term in the expansion of ${\left(\gamma_{g}\right)}^{2}$ was 500. There are subtle differences between the results of QP and SP; however, for the most part, only minor differences have been observed in decimal place accuracy. The CMAA-SSQ provides a more equitable case mix than CMAA-LIN, but less equitable than CMAA-EQ. Hence, it lies between the CMAA-EQ and CMAA-LIN approaches. This conclusion is justified by the values presented in Column 7 in Table 2, Table 3 and Table 4.

In Table 3, the alternative CMAA-LIN approach is demonstrated. The resulting case mixes are quite different from those exhibited in Table 2. In general, the CMAA-LIN tends to limit the number of patient types exhibiting a change. The CMAA-EQ is more equitable but realises a bigger alteration than the CMAA-LIN, resulting in fewer patients treated overall. Hence, the CMAA-LIN produces case mixes with a greater number of patients.

**Insights**: Changes to the specified case mix were discussed above, and in those numerical tests, we assumed the patient sub mixes implied by the original starting case mix are maintained. Hence, whatever proportions are inherent therein are not altered. In many situations, we would expect the impact to other patient types to be of the same order of magnitude of the original alteration. However, some alterations of the case mix permit higher or lower increases (decreases) to be realized. These impacts are not directly proportional to the scale of the original alteration. This means that there may be some latent unused capacity relative to the original case mix.

Specific changes to a particular patient subtype are also worth considering. A similar process and model can be posed for this situation. The exact details are shown below (Algorithm 2):
**Algorithm 2.** Impact Assessment (SubTypes)Step 1. Select a case mix $n^{2}$, and designate targets ${\hat{n}}_{g,p}^{2}=n_{g,p}^{2}$.Step 2. Select a change of δ units in one patient subtype $\left(g^{*},p^{*}\right)$, i.e., $\left(\delta ,g^{*},p^{*}\right)|-{\hat{n}}_{g^{*},p^{*}}^{2}\leq \delta \leq {\bar{n}}_{g^{*},p^{*}}^{2}-{\hat{n}}_{g^{*},p^{*}}^{2}$ where δ≠0 and ${\bar{n}}_{g,p}^{2}$ is the upper bound on the number of patients of subtype (g,p) that are treatable.Step 3. Solve the model below to obtain a new case mix $n^{2}$$.\;\mathrm{Report}\;\mathrm{the}\;\mathrm{differences}\;\gamma_{g,p}$.   Minimize Δ×Z   where $Z=\underset{g\in G}{\sum }\underset{p\in P_{g}}{\sum }{\left(\gamma_{g,p}\right)}^{2}$ or $Z=\underset{g\in G}{\sum }\underset{p\in P_{g}}{\sum }\gamma_{g,p}$ or Z=λ
(10)   Subject to:   
$n_{g^{*},p^{*}}^{2}={\hat{n}}_{g^{*},p^{*}}^{2}+\delta$(11)   
$\gamma_{g^{*},p^{*}}=0$ and $\gamma_{g,p}=\Delta \times \left(\frac{{\hat{n}}_{g,p}^{2}-n_{g,p}^{2}}{{\bar{n}}_{g,p}^{2}}\right)\forall g\in G\backslash \left\{g^{*}\right\},p\in P_{g}\backslash \left\{p^{*}\right\}$(12)   $\Delta \times n_{g,p}^{2}\leq \Delta \times {\hat{n}}_{g,p}^{2}$  $\forall g\in G\backslash \left\{g^{*}\right\},p\in P_{g}\backslash \left\{p^{*}\right\}$(13)   
$n_{g,p}^{2}\geq {\hat{n}}_{g,p}^{2}-\Delta \times \lambda {\bar{n}}_{g,p}^{2}$ $\forall g\in G\backslash \left\{g^{*}\right\},p\in P_{g}\backslash \left\{p^{*}\right\}$(CMQ-EQ only)
(14)   $\gamma_{g,p}\geq 0$   $\forall g\in G,p\in P_{g}$(15)   $n_{g,p}^{2}\geq \mu_{g,p}^{2}n_{g}^{1}$   $\forall g\in G\backslash \left\{g^{*}\right\},p\in P_{g}$(16)   Constraints (A2)–(A6) from Appendix A

It is necessary to point out that in constraint (16) sub mix proportions are enforced for all patient groups except $g^{*}$.

**Example 2.**  *Consider the case mix* $n^{2}=\left(\left[3.97,\;1.7],\;[48.82],\;[5.11,\;8.17,\;7.15],\;[10.22],\;[28.38\right]\right)$ *associated with $n^{1}$* *in Example 1. If subtype T1–1 was chosen and an alteration of 5 was selected, then ${\hat{n}}_{1,1}^{2}\to 8.97$. For the CMAA-EQ option, the following case mix is obtained, $n^{2}=$((8.97, 1.32), (48.54), (4.99, 7.99, 6.99), (9.9), (28.16)). Associated with that case mix are the following alterations, ((5,−0.38),(−0.277),(−0.12.−0.18,−0.16),(−0.32),(−0.22)). Hence, the new case mix is $n^{1}=$(10.29, 48.54, 19.96, 9.9, 28.16) and ℕ=116.85.*

### 2.3. Case Mix Comparisons

In this section, multi-criteria decision-making theory is adapted to help support hospital CMP. A multi-criteria decision support tool (MCDST) is also proposed. In hospital CMP, the identification of a single case mix solution that meets certain conditions is important and has been sought in previous articles. The identification of alternative case mix solutions is valuable and constructive. As shown in Section 2.2, further analysis of a single case mix solution is worthy. It also makes sense to start with a single case mix solution and to suggest alterations. In the previous section, we considered how to assess the impact of any change, and a mathematical model was proposed for that purpose. It produces a second case mix and provides the decision-maker with two case mixes to compare. However, this raises the question, how does a decision-maker determine which is preferable?

In multi-criteria CMP, it is essential to identify an assortment of potential solutions, namely a Pareto frontier of alternatively optimal case mix solutions. As there are multiple conflicting objectives, no single solution exists that simultaneously optimizes each objective. All Pareto optimal solutions are considered equally good without further preference information. A solution is called Non-Dominated, Pareto Optimal, or Pareto efficient, if none of the objective functions can be improved without degrading some other objective values. To obtain a Pareto frontier, there are various techniques, the basis of which is the solution of an underlying mathematical optimization model with multi-objectives.

In Burdett et al. [2], a high dimensional CMP problem was solved, and tens of thousands of Pareto optimal case mix solutions were found. How a single solution could be chosen by hospital planners, managers, and executives was identified as challenging. To handle this dilemma, it seems reasonable to provide a means of scoring an individual case mix solution, and second to provide a means of critiquing two case mixes, providing insight into which is better or worse. This aligns well with the task discussed in Section 2.2. To the best of our knowledge, previous articles have not formally scored patient case mix solutions, nor provided techniques to compare competing case mix solutions purely based on the number of patients treated.

#### 2.3.1. Pareto Optimal Case Mix

There is a set of feasible case mixes, hereby denoted N, that the system can facilitate and treat. It is a subset of ${\mathbb{R}}^{\left|G\right|}.$ Set N can be partitioned into a set of non-dominated case mixes (i.e., $N^{*}$) and a set of dominated case (i.e., $N^{d}$). The non-dominated case mixes are Pareto optimal solutions to a multicriteria case mix optimization problem whereby $F\left(n_{g}\right)$ is independently maximized. In this article, we assume that $F\left(n_{g}\right)=n_{g}$.

Regarding set $N^{*}$, it is worth noting the following: All Pareto optimal case mixes are considered equally good. In a Pareto optimal case mix, none of the $n_{g}$ can be improved except by degrading some other $n_{g}$ values. Dominance is a fundamental concept in MCO. Case mix n is said to dominate case mix $n^{\prime }$ (i.e., $n≽n^{\prime }$) if it is better in at least one objective, and the same as or better in the other objectives (i.e., $F\left(n_{g}\right)\geq F\left(n_{g}^{\prime }\right)\;\forall g\in G$ and $\exists g\in G|F\left(n_{g}\right)\rangle F\left(n_{g}^{\prime }\right)$). A Pareto optimal case mix is not dominated by any other case mix. A case mix is Pareto optimal if $∄\left(n_{1}^{\prime },n_{2}^{\prime },\ldots ,n_{\left|G\right|}^{\prime }\right)\in N^{d}$ where $F\left(n_{g}^{\prime }\right)\geq F\left(n_{g}\right)\;\forall g\in G$ and $\exists g\in G|F\left(n_{g}^{\prime }\right)\rangle F\left(n_{g}\right)$.

In the following sections, we demonstrate how to define a score for each case mix n∈N. We also demonstrate how to compare any pair of case mix $\left(n,n^{\prime }\right),$ where $n,n^{\prime }\in N|n\neq n^{\prime }$.

#### 2.3.2. Scoring Case Mix

Every case mix n can be compared to the ideal (a.k.a., utopia) and anti-ideal (a.k.a., nadir) case mix. The ideal case mix denoted $\bar{n}$ occurs when each patient type achieves its upper bound. The anti-ideal is the case mix with zero patients of each type, which is hereby denoted $\underline{n}$. The proximity of n to $\bar{n}$ is an important indicator of progress for obvious reasons and can be used within a scoring mechanism. The proximity can be computed according to various metrics, and a relative progress score may be useful as defined below:

(17) $${\mathrm{score}}_{1}\left(n,k\right)=100\times \left(1-{\mathrm{proximity}}_{k}\left(n,\bar{n}\right)/{\mathrm{proximity}}_{k}\left(\underline{n},\bar{n}\right)\right)$$

(18) $${\mathrm{score}}_{2}\left(n,k\right)=100\times {\mathrm{proximity}}_{k}\left(\underline{n},n\right)/{\mathrm{proximity}}_{k}\left(\underline{n},\bar{n}\right)$$

To evaluate (17) and (18) we assume that ${\mathrm{proximity}}_{k}\left(n^{A},n^{B}\right)={\left\|\Delta n\right\|}_{k},$ where $\Delta n_{g}=\left(n_{g}^{A}-n_{g}^{B}\right)/\epsilon_{g}$. If k=1 is chosen, the one-norm is applied and ${\left\|\Delta n\right\|}_{1}=\underset{g\in G}{\sum }\left|\Delta n_{g}\right|$. If k=2 is chosen, the two-norm is applied and ${\left\|\Delta n\right\|}_{2}={\left(\underset{g\in G}{\sum }{\left(\Delta n_{g}\right)}^{2}\right)}^{1/2}$. Both progress scores provide values in the range [0,100] and have value 100 when $n=\bar{n}$ and the value zero when $n=\underline{n}$. The denominator is a scaling mechanism and represents the distance between the ideal and anti-ideal. As some patient types may have vastly different orders of magnitude, the proximities should also be normalised, to maintain objectivity. The default value for $\epsilon_{g}$ is ${\bar{n}}_{g},$ and this ensures each value in the summation does not exceed one. The value $\epsilon_{g}$ may also be seen as a user-defined measure of significance for patients of type g. The range for $\epsilon_{g}$ is therefore $\left(0,{\bar{n}}_{g}\right].$ Notably, when $\epsilon_{g}$ is small, differences are amplified greatly.

**Final Remarks.** An alternative course of action is to scale everything first, that is, to set define $\bar{n}=\left(1,1,\ldots ,1\right)$, $\underline{n}=\left(0,0,\ldots ,0\right),$ and to set $n=\left(\frac{n_{1}}{{\bar{n}}_{1}},\frac{n_{2}}{{\bar{n}}_{2}},\ldots ,\frac{n_{\left|G\right|}}{{\bar{n}}_{\left|G\right|}}\right)$. It is easy to show that the same answer is obtained. Either the 1-norm or 2-norm are reasonable to apply. The former (i.e., rectilinear distance) is a measure of absolute difference in each of the |G| dimensions. The second (i.e., Euclidean) is a direct distance measure of separation. Interestingly, when the 1-norm is applied, ${\mathrm{proximity}}_{1}\left(\underline{n},n\right)+{\mathrm{proximity}}_{1}\left(n,\bar{n}\right)=\mathrm{proximity}\_1\left(\underline{n},\bar{n}\right)$. Consequently, $score_{1}=score_{2}$.

**Figure 1:**
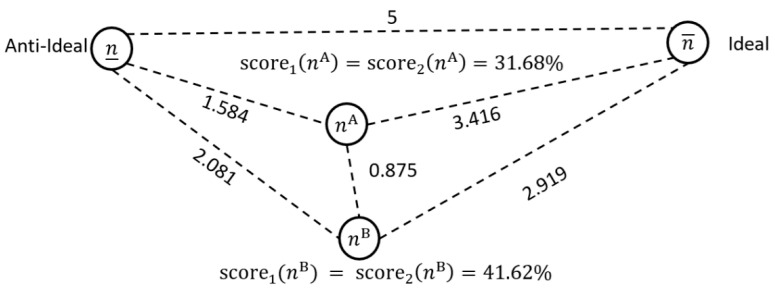
Comparing case mix solutions using the 1-norm.

**Figure 2:**
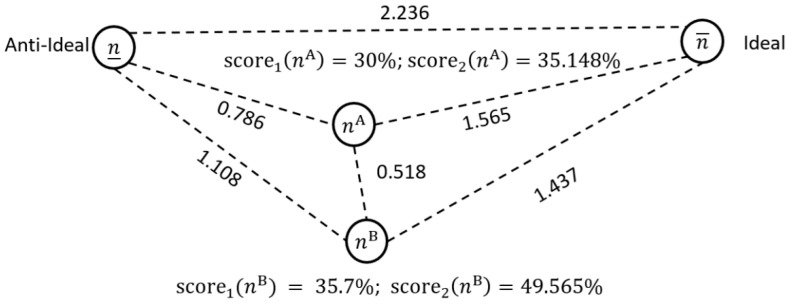
Comparing case mix solutions using the 2-norm.

**Example 3.**  *Let us consider two case mixes with five patient types. These are, respectively, *$n^{A}=$ *(5.68, 48.82, 20.43, 10.22, 28.38) and* $n^{B}=$ *(16.46, 71.67, 11.79, 10.59, 24.39). Notably, neither of these case mixes dominate the other. Case mix* $n^{A}$ *is better for two groups and* $n^{B}$ *for three. If the ideal solution is* $\bar{n}=\left(25.18,\;89.79,\;65.48,\;105.05,\;70\right)$*, the proximities and scores shown in Figure 1 and Figure 2 result. Evidently, the second case mix is further from the anti-ideal and closest to the ideal. The second case mix has higher numbers of patient type G1 and G2, and similar numbers of G4 and G5. Only in patient type G3 is it more noticeably deficient. The scaled distances of 0.875 and 0.518, respectively, indicate some difference and dissimilarity. Relative to their proximity to the ideal and anti-ideal, case mix A and B are closer to each other. The low*$score_{1}$*values show that both case mixes are far from the ideal. The*$score_{2}$*values are slightly higher, indicating a greater distance from the anti-ideal.*

**Final Remarks**. The significance level is highly influential in the assessment, yet inherently subjective. Two case mixes may have the same proximity yet be placed in a totally different parts of the objective space. In those circumstances, these approaches are unhelpful for making any type of judgement. It seems that an iterative approach, involving an alteration of the level of significance, may be necessary, to build up some understanding of the merit of one case mix over another. For instance, it may be necessary to identify if small changes to the level of significance change the resulting comparison, and how those changes skew the proximity.

**Figure 3:**
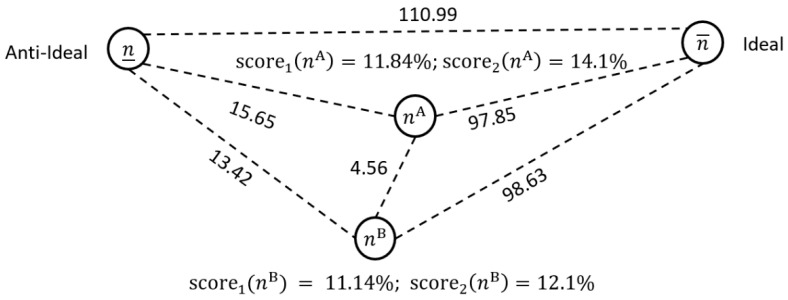
Comparing case mix solutions using the 2-norm and the given ϵ vector.

**Demonstrative Example (Cont’d):** If we assume that ϵ=(15, 25,2,1,5), then the resulting scores and proximities are shown in Figure 3. We can see that case mix one is not that different from case mix two, except in group G3. The distance between the case mix (i.e., of 4.56) is bigger than previously shown in Figure 2, but relative to the distance between the ideal and non-ideal, it is comparatively smaller. The results in Figure 3 are different to those found in Figure 1 and Figure 2 because $n_{3}^{A}\gg n_{3}^{B}$. As such, the first case mix has a higher score.

#### 2.3.3. Quantifying Similarity and Dissimilarity

In the previous section, we assumed proximity to the ideal equates to achievement. We also implied indirectly that proximity equals similarity. For instance, if two case mixes are close, then they must be similar. This notion may be challenged, particularly when levels of significance are incorporated. A more rigorous way to perform a comparison, and to judge similarity/dissimilarity, may be achieved using Definition 1 and its corollary. Definition 1 is based upon the e-dominance principles of Laumanns et al. [27], later adapted, for instance, by Hancock et al. [28]. When using the concept of e-dominance, the user is expected to provide a value that represents the minimum amount of change in an objective that is considered significant. In other words, the user defines what a significant difference is. In some scenarios, a difference of one unit implies significant difference, but in others, it would not. The application of the e-dominance principle is akin to partitioning the objective space into regions.

**Definition 1.**  *Two case mix* $n^{A},n^{B}\subset {\mathbb{R}}^{\left|G\right|}$*, are similar if* $\left|n_{g}^{A}-n_{g}^{B}\right|\leq \epsilon_{g}\;\forall g\in G$*. If* ∃g∈G| $\left|n_{g}^{A}-n_{g}^{B}\right|>\epsilon_{g}$ *then we can conclude significant difference.*

**Corollary 1.**  *If case mix solutions are not similar, then significant trade-offs must be realized if one solution is selected over another.*

**Corollary 2.**  *Around any solution* n *, the boundary of the region of similarity-dissimilarity can be identified, for instance by identifying all solutions* n *such that* $\epsilon_{g}\leq \left|n_{g}-n_{g}^{\prime }\right|\leq \left(1+\lambda \right)\epsilon_{g}\;\forall g\in G$.

Definition 1 is used to identify significant differences between case mixes; however, they may be similar in various ways. As such, the level of similarity (LOS) is a concept worth defining. A possible measure of the level of similarity between two case mixes, $n^{A},n^{B}\subset {\mathbb{R}}^{\left|G\right|}$ is the number of groups that have similar n value. Formally, $SIM_{1}=100\times \left(\left|G^{\prime }\right|/\left|G\right|\right)$ where $G^{\prime }=\left\{g\in G|\left|n_{g}^{A}-n_{g}^{B}\right|\leq \epsilon_{g}\right\}$. Naturally, the level of dissimilarity ($DISIM_{1}$) becomes $100-SIM_{1}$ or $100\times \left(\left|G^{\prime \prime }\right|/\left|G\right|\right)$ where $G^{\prime \prime }=\left\{g\in G|\left|n_{g}^{A}-n_{g}^{B}\right|\rangle \epsilon_{g}\right\}$. Various Joint measures of similarity and dissimilarity can be defined as follows:

(19) $$DISIM_{2}=\sum_{g\in G}\max\left(\left|n_{g}^{A}-n_{g}^{B}\right|-\epsilon_{g},0\right)/\epsilon_{g}$$

(20) $$DISIM_{3}=\sqrt{\sum_{g\in G}\max\left({\left(\left|n_{g}^{A}-n_{g}^{B}\right|-\epsilon_{g}\right)}^{2},0\right)/{\left(\epsilon_{g}\right)}^{2}}$$

These measures penalize differences outside the tolerance and, in principle, are similar concepts. Bigger values mean greater dissimilarity and smaller, less. The differences are also scaled to ensure different orders of magnitude do not skew the results. As mentioned earlier, proximity is a possible measure of similarity and dissimilarity. We can then define a fourth measure, $DISIM_{4}\left(k\right)={\mathrm{proximity}}_{k}\left(n^{A},n^{B}\right)$.

**Example 4.**  *If we compare case mix* $n^{A}=\left(5.68,\;48.82,\;20.43,\;10.22,\;28.38\right)$ *and* $n^{B}=\left(16.46,\;71.67,\;11.79,\;10.59,\;24.39\right)$ *and define* ϵ=(2.5, 9, 6.5, 10.5, 7)  *then, only in patient type G4 and G5 is there a lack of significant difference. Hence, we could say that the level of similarity is 40% (i.e., 2/5) and the level of dissimilarity is quite high at 60%. The other metrics are shown in* Table 6 *. In that table, other* ϵ *vectors have also been evaluated to demonstrate how the comparison is skewed.*

Table 1 shows there can be significant differences between the $DISIM_{2}$ and $DISIM_{3}$ values as the level of dissimilarity increases. Generally, $DISIM_{2}$ results in larger values than $DISIM_{3}$. The $DISIM_{1}$ metric performs very differently from the others.

The third ϵ vector is notable because its $DISIM_{1}$ value is quite small, yet its $DISIM_{2-4}$ values are higher than the fourth ϵ vector, which has higher dissimilarity. The cause of this disparity are the outputs for group G2. The difference in outputs is very large (i.e., 71.67−48.82 = 22.85), and the level of significance is small (i.e., 2).

#### 2.3.4. Comparing Case Mix

It is desirable to make a judgement upon which case mix, say $n^{A}$ or $n^{B}$, is most preferable. Previously defined scoring mechanisms are useful to some extent but only judge merit based upon the proximity to the ideal. Determining a more transparent approach that can clarify the exact nature of the trade-offs is worthy of consideration.

**Figure 4:**
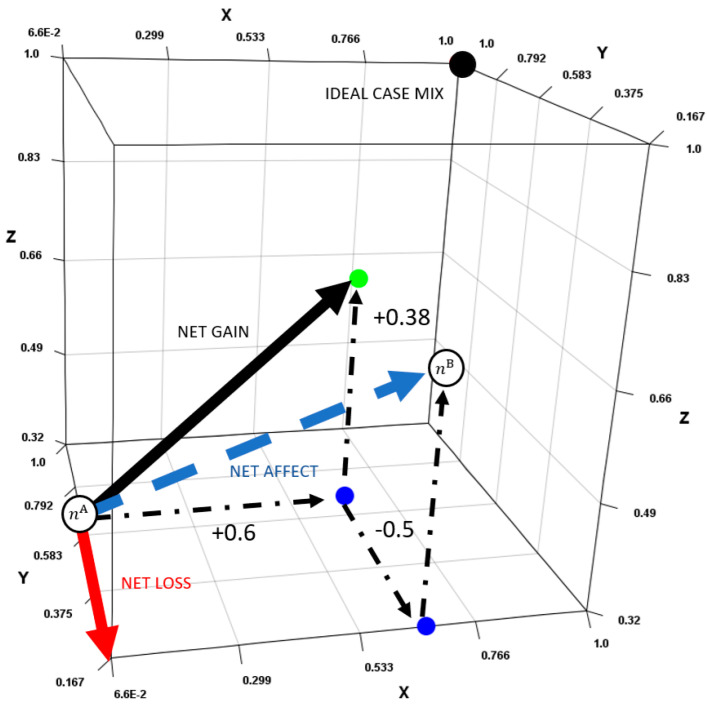
Comparing case mix (1,20,16) and (10,5,35).

In this section, we develop an approach based upon the idea described in Definition 2. We propose that differences between the case mixes are partitioned into gains and losses. An example is shown in Figure 4. The gains and losses can then be aggregated using the concept of a resultant vector. This produces two values that can be easily compared to make a judgement. Compared to an approach involving |G| comparisons, the new approach is more transparent and user-friendly. Our approach is primarily defined to critique differences in the number of patients of each type and to make judgements relative to that metric. Other metrics can also be compared, notably, financial metrics. These can be aggregated into a single “dollar” value but may be less well-suited to this type of analysis.

**Definition 2.**  *Given two case mix solutions* $n^{A},n^{B}\subset {\mathbb{R}}^{\left|G\right|}$ *, we can say that one case mix is preferable if the ratio of the net gain* $G^{+}$ *to the net loss* $G^{-}$ *, namely* $R=\frac{G^{+}}{G^{-}},$ *is not roughly one or if the difference* $\left(G^{+}-G^{-}\right)$ *is not zero. In other words, the next gain is not roughly the same as the net loss.* *The net gain is computed as* $G^{+}={\left\|V^{+}\right\|}_{k}$ *for* k=1 *or* k=2 *, where* $V^{+}=\underset{g\in G|\delta_{g}\rangle 0}{\sum }\delta_{g}{\tilde{u}}_{g}$ *is the resultant “gain” vector. Furthermore, the net loss is computed as* $G^{-}={\left\|V^{-}\right\|}_{k},$ *where* $V^{-}=\underset{g\in G|\delta_{g}<0}{\sum }\delta_{g}{\tilde{u}}_{g}$ *is the resultant “loss” vector. It is important to recognize that* ${\tilde{u}}_{g}$ *are “unit” basis vectors and either* $\delta_{g}^{A,B}=\left({\hat{n}}_{g}^{B}-{\hat{n}}_{g}^{A}\right)$ *where* ${\hat{n}}_{g}=\left(n_{g}-{\underline{n}}_{g}\right)/\left({\bar{n}}_{g}-{\underline{n}}_{g}\right)$ *or* $\delta_{g}^{A,B}=\left(n_{g}^{B}-n_{g}^{A}\right)/\epsilon_{g}$ *. Lastly, if* $G^{-}=0$ *, the ratio cannot be used, and it is best to base the comparison on the difference.*

**Corollary 3.**  *When using* $\delta_{g}=\left(n_{g}^{B}-n_{g}^{A}\right)/\epsilon_{g},$ *we can say that one solution is “significantly” better.*

**Corollary 4.**  *If* $G^{+}<G^{-}$ *then* $n_{g}^{A}≽n_{g}^{B}$*, where “*≽*” is used to signify “better than”. Similarly, if* $G^{+}>G^{-}$ *then* $n_{g}^{B}≽n_{g}^{A}$.

**3D Example [a].** Consider case mix (1,20,16) and (10,5,35) (i.e., Figure 4) where x∈[0,15], y∈[0,30], z∈[0,50]. These case mixes have achievement levels of 30.6% and 45.36%, respectively, so we would expect case mix two to be most preferable. The normalized points are (0.066,0.666,0.32) and (0.666,0.166,0.7). The net differences are (+9,−15,+19) but after normalization, (+0.6,−0.5,+0.38). The net gain is 0.71, the net loss is 0.5, and the ratio is 1.42. Hence, the second case mix is convincingly most preferable as the net gain significantly exceeds the net loss. Visually, we can see that the net effect is not directly towards the ideal, but to the right of it.

**3D Example [b].** Consider case mix (14.87,15,0) and (0, 16.25, 50), where x∈[0,15], y∈[0,30], and z∈[0,50]. Both case mixes are equally positioned from the ideal, at 1.11807, with an achievement level of 35.4483%. It is, hence, not clear which case mix is most preferable based upon that metric. Distance from the ideal is 1.11 (i.e., 64.1%) and 0.966 (i.e., 55.78%), respectively, indicating case mix one is further away from the non-ideal solution. The normalized points are (0.9913, 0.5, 0) and (0, 0.5417, 0.8). The net differences are (−14.87,+1.25,+40) and after normalization, (−0.9913,+0.0417,+0.8). The net gain is 0.801, the net loss is 0.9913, and the ratio is 0.8081. Hence, the first case mix is preferable as the net loss exceeds the net gain.

**Higher-Dimensional Example**: When comparing case mix one (5.68, 48.82, 20.43, 10.22, 28.38) to case mix two (16.46, 71.67, 11.79, 10.59, 24.39), we note that case mix 2 has 21.37 more patients. There is a direct gain of 34 patients (i.e., spread across G1, G2, G4), and a loss of 12.63 (i.e., spread across G3 and G5). The differences are as follows: (10.78, 22.85,−8.64, 0.37,−3.99). As such, $V^{+}=\left(0.43,\;0.25,\;0,\;0.0032,\;0\right)$ and $V^{-}=\left(0,\;0,\;0.132,\;0,\;0.057\right)$. Hence, the net gain is $G^{+}=0.498$ and the net loss is $G^{-}=0.144$ with a ratio of R=3.465. The second case mix is preferable as the net gain exceeds the net loss.

It is foreseeable that a decision-maker may also be interested in trade-offs occurring between some patient types and not others, which are deemed unimportant for one reason or another. The proposed approach can easily be modified to assess net gains and losses only in those types, as opposed to the automatic inclusion of all patient types. An assessment where multiple comparisons are performed separately to identify if comparatively higher amounts of deterioration in one or more patient types occurs is also possible.

**Demonstrative Example (Cont’d)**: Reconsidering the previous case mixes $n^{A}=\left(5.68,\;48.82,\;20.43,\;10.22,\;28.38\right)$ and $n^{B}=\left(16.46,\;71.67,\;11.79,\;10.59,\;24.39\right)$, let us assume that trade-offs between patient type G3, G4, and G5 are important. The considered differences are (−8.64, 0.37,−3.99). As such, $V^{+}=\left(0,\;0.00352,\;0\right)$ and $V^{-}=\left(0.132,\;0,\;0.057\right)$. Hence, the net gain is $G^{+}=0.00352$ and the net loss is $G^{-}=0.144$ with a ratio of R=0.245. Therefore, the first case mix is preferable as the net loss exceeds the net gain.

## 3. Putting Theory into Practice

To facilitate CMP activities, a prototype PDST was created (Burdett et al. [14]). The tool permits users to determine the maximum number of patients that may be treated over time, subject to case mix and time availability constraints. This task is performed by solving the model shown in Appendix A. Given user-defined targets, a best-fit case mix is also obtainable by solving a non-linear variant of the model. The feasibility of a specified case mix can also be checked within the PDST.

**Figure 5:**
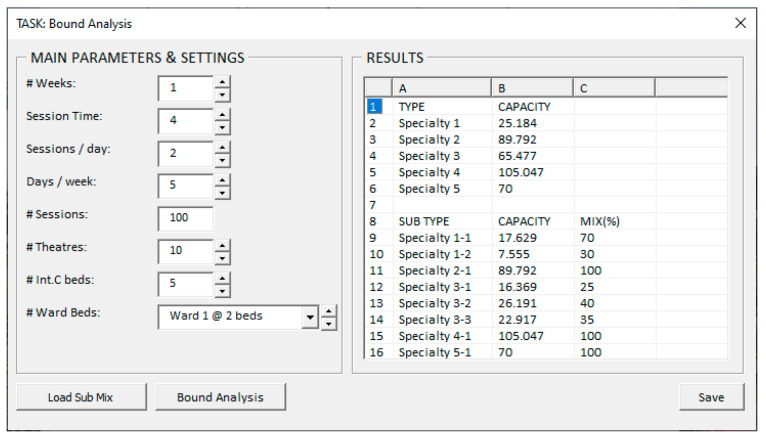
GUI to perform a bound analysis.

The two tasks discussed in previous sections—namely case mix alteration assessment and case mix comparison—have been added to the PDST, and suitable graphical user interfaces (GUI) have been constructed. Both tasks, however, rely upon the upper bound for each patient type. This bound describes the maximum flow of patients of type g that can be realised if the resources of the entire hospital were used to process only that patient type. The window shown in Figure 5 is provided for users to activate this assessment. The “Bound Analysis” button activates the solution process, involving the solution of the CMQ model |G| times. For patient type g∈G, the case mix is set as $\mu_{g}^{1}=1$ and $\mu_{g^{\prime }}^{1}=0\;\forall g^{\prime }\in G\backslash \left\{g\right\}$. The results shown in the right pane are generated and displayed progressively but may also be shown all at once.

### 3.1. Case Mix Alteration Assessments

**Figure 6:**
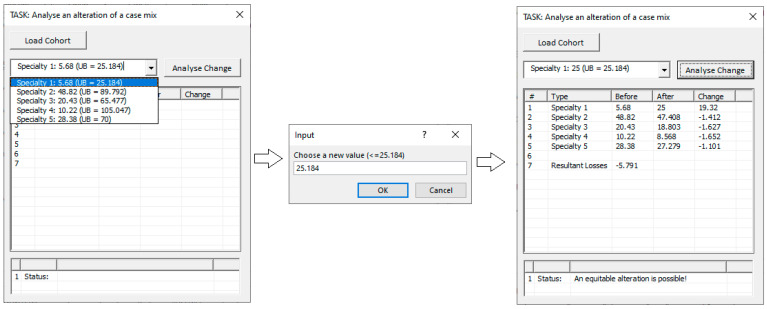
GUI to facilitate case mix alteration assessment.

To alter a case mix and to analyse the effect, the GUI shown in Figure 6 has been created. The first step is to specify the current case mix using the “Load Cohort” button. The combo box summarises the different patient types, their current $n_{g}^{1}$ value, plus for reference purposes, the upper bound ${\bar{n}}_{g}^{1}$. Any patient type can be selected and altered using the drop-down selection mechanism. The “Analyse Change” button informs the user to enter a δ value such that $-n_{g}^{1}\leq \delta \leq {\bar{n}}_{g}^{1}$. If satisfactory, the CMQ model is then solved. In the bottom ListView, the results are displayed. The before-and-after values are shown, plus the required changes. The user is asked whether the results are accepted or rejected. Further alterations to other patient types are then permitted to be analysed. In other words, a hierarchical assessment is facilitated.

**Figure 7:**
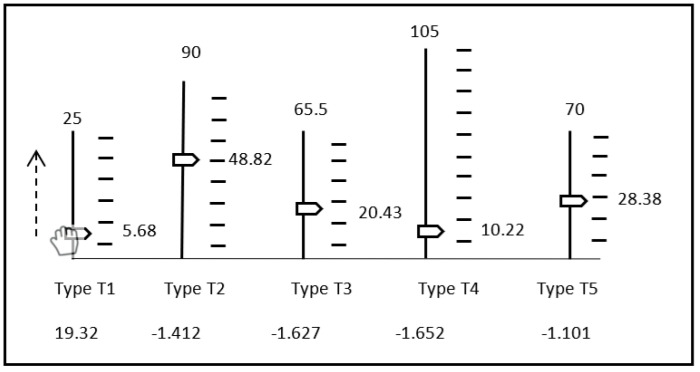
An alternative interface (of sliders) for users to manipulate.

Figure 7 shows an alternative approach yet undeveloped, involving the manipulation of a set of sliders. Each slider is for a specific patient type. The current value for each patient type is shown as marker on a bar, whose height is relative to the upper bound. A single patient type can then be selected easily and incremented and decremented to whatever value the user likes. The change to other patients can then be shown immediately, and the overall effect can be witnessed. For instance, changes in the height of each marker can be visualised. In a typical hospital scenario, there are no more than 20–30 major patient types, and those can fit within one screen. If there are more, a chart of this nature may be too congested/busy, however.

### 3.2. Case Mix Comparisons

**Figure 8:**
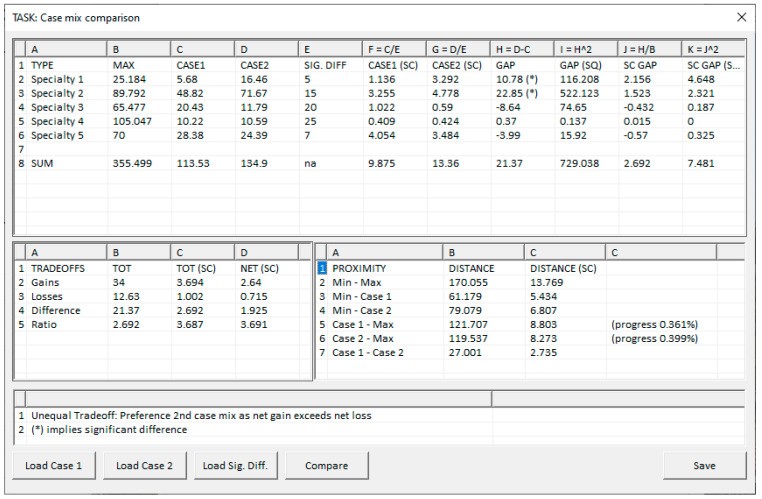
GUI to facilitate case mix comparisons.

To critique two case mixes, the GUI displayed in Figure 8 has been developed. It is worth noting that “SQ” is an abbreviation for a squared value and “SC” for a scaled value. Also, “Min” refers to the anti-ideal solution and “Max” the ideal. In that GUI, the first step is to load two candidate case mixes. The next step is to choose a level of significance for each patient type. If none is selected, then the present upper bounds are used. The “Compare” button activates the assessment and applies the calculations from Section 2.3, specifically those associated with Definition 2. The GUI currently shows all calculations involved. This highly detailed output is for experts and is extraneous to the average end user. In future versions, this information could possibly be hidden, accessible only in reports or by direct query.

Figure 8 shows a situation where a level of significance has been defined. In column H, differences exceeding $\epsilon_{g}$ are highlighted using (*). The level of significance that has been used has amplified the gains and losses and placed both case mixes closer to the ideal.

## 4. COVID-Inspired Case Study

COVID-19 is an infectious disease (a.k.a., coronavirus) that causes mild to moderate respiratory illness in most people. Originating at the end of 2019, it has been “in play” for about 4 years. In the early stages, many countries were unable or unwilling to quarantine this virus, and consequently, its spread throughout the world was assured.

Most hospitals have been seriously impacted by COVID-19, and the virus has interfered with the day-to-day operations of most. For instance, this virus has caused many additional representations, and many of those patients have been seriously ill. To handle that demand, many elective surgeries were postponed, and many wards and beds have been repurposed. Intensive care facilities, used for ventilating patients mechanically, have been particularly stretched.

This article’s methods are well-suited to analyzing the impact of COVID-19 or any other virus or medical disaster on the capacity of a hospital. To demonstrate that assertion, let us reconsider the earlier scenario with five intensive care beds, 10 operating theatres, five recovery wards, and five patient types. Let us now consider a longer time frame of 2 months and the arrival of additional COVID-19 patients who are admitted to the hospital for treatment. We will now identity the effect on the current case mix of 113.53 patients per week where $n^{1}$ = (5.68, 48.82, 20.43, 10.22, 28.38). The COVID-19 patients are defined as a sixth patient type, namely G6. Amongst that cohort, various subtypes can be defined. We define four subtypes according to severity of illness at presentation (Gulsen [29]). The clinical pathway and average length of stay for each subtype is show in Table 7. These values were adapted from the data summarized in Vekaria et al. [30] and Whitfield et al. [31]. To cope with anticipated demand, a new ward (i.e., Ward 6) has been set up in our example and several current wards (i.e., Ward 1 and 5) have been re-purposed as shown in Table 8. Table 9 provides revised bounds and other relevant patient information. All COVID-19 patients are assumed to be kept in isolated wards to restrict transmission.

For this analysis, we have added surgery times to ward length of stay, as beds are acquired before surgery begins. Subject to the proportional case mix (0.05, 0.43, 0.18, 0.09, 0.25, 0) the treatable cohort (i.e., hospital capacity) is 908 patients of the following types $n^{1\left(\mathrm{orig}\right)}=$ (45.41, 390.54, 163.48, 81.74, 227.06, 0). After the hospital’s layout is changed, the capacity to treat non-Covid patients is not altered, even though fewer wards and beds are available. This occurs because some restrictions have been relaxed. For instance, ward W4 has been permitted to treat more patient types.

Let us now consider what case mix alterations are required if COVID-19 patients of type T6 must be treated. Let us also consider whether the hospital reconfiguration is sufficient to meet the demand, and whether further wards should be repurposed. Pre-analysis shows that no more than 21.5 COVID-19 patients can be treated per week, given the sub mix (0.45, 0.35, 0.15, 0.5). Hence, we analyze the effect of T6 patients in the range (0, 172].

Table 10 shows that the hospital can meet COVID-19 patient demands of 12–13 patients/week quite easily and has some capacity to spare. As the number increases further, the original cohort of different patient types is greatly affected, and maintaining an equitable decrease reduces overall outputs considerably. However, some of the original cohort can still be treated. The exact number is shown in brackets in column 3. Table 11, however shows that if equity does not matter, then the hospital can still treat a high number of patients, relative to “pre-Covid” times. However, some patient types can be exploited, like G5. The model found that reducing G5 admissions provides the capacity to treat most of the original cohort and the new covid patients.

Table 12 shows the SSQ approach and demonstrates the “in-between” approach, which is somewhat more equitable than the CMAA-LIN variant. Types G1 and G5 are reduced the most.

**Final Remarks:** The above analysis could be repeated for any layout alteration that is being considered and for any-sized hospital. An iterative approach considering a sequence of changes is also appropriate. In the above situation, if we later decided that more than 21.5 COVID-19 patients were to be treated per week, another reconfiguration would have to be envisaged and analyzed.

## 5. Conclusions

This article proposes practical analytical techniques to support hospital case mix planning supplementing methods we have developed and applied to real hospital settings over the last 10 years. Hospital case mix planning is an emerging field that examines the implications of treating different patient cohorts and aims to guide hospital management in determining which patient cohort should be prioritized over others. Ultimately, the choice of case mix is synonymous to choosing a single Pareto optimal solution in a multi-criteria objective space. Though the idea of CMP is appealing, the reality is that most hospitals do not apply any formal CMP techniques. They operate dynamically and treat patients as they emerge, considering severity of illness to prioritize one patient type over another. The master surgical schedule is also influential, chosen to satisfy surgeons and their availability, at the expense of all other considerations.

To advance the state of the art, we explored how changes to an existing patient case mix can either free up capacity for other patient types or reduce it. Our mathematical optimization model generates a solution that quantifies the impact on other patient types, identifying how much additional patient flow can be treated, or which patient types need to be reduced, and by what extent. Our approach naturally provides hospital planners with a sensitivity analysis that can inform and guide an iterative approach to achieve the most optimal case mix.

We also developed some useful techniques to assess, compare, and critique competing case mixes. A score based upon proximity to the ideal was first proposed. Measures of similarity and dissimilarity were then proposed. These are based upon the concept of e-dominance. Last, an approach to measure trade-offs and to aggregate those into a net gain and net loss were developed. These statistics permit an end user a more transparent means of judging overall merit and a way to judge which case mix is superior.

In summary, the proposed approach forces an end user to clarify and disclose their belief around the value of treating patient types in different numbers and a means to adapt their beliefs or requirements. In this article, the total flow of patients of each type was treated as the main objective. However, other objectives may also be considered, like reimbursement or revenue. Regarding the uptake of these methods, graphical user interfaces were proposed, implemented, and tested. The resulting decision support tool looks viable, and further developments are being considered. In future research, we plan to explore how hospital layout and functionality influence the case mix landscape and how these elements can be optimized to align with the objectives of healthcare managers and planners. We are particularly interested in strategically reorganizing treatment areas in local hospitals to enhance operational flexibility. Our focus is on designing hospitals that can efficiently accommodate a variety of selected patient case mixes.

The main limitation of our approach is the assumption of deterministic parameters. We plan to create a more sophisticated stochastic approach in the future.

## Figures and Tables

**Table 1:** Patient type information.

Type	${\bar{n}}^{1}$	# Subtypes	Subtype	Subtype Mix (%)	(ic, sur, postop) Time (# h)	Wards Used
T1	25.18	2	T1–1	70	(0, 1.2, 17.86)	W1
			T1–2	30	(6, 1.25, 8.35)	W1, W2
T2	89.79	1	T2–1	100	(0, 2.4, 16.31)	W1, W2, W5
T3	65.48	3	T3–1	25	(0, 6.5, 12.94)	W3
			T3–2	40	(0, 4.56, 12.39)	W3
			T3–3	35	(0, 7.6, 5.54)	W3
T4	105.05	1	T4–1	100	(0, 3.4, 18.99)	W4
T5	70	1	T5–1	100	(12, 4.1, 22.81)	W4, W5

**Table 2:** Application of “CMAA-EQ” model for the selected case mix.

	Revision	δ	Case Mix Alterations	New Case Mix	ℕ	Impact	λ
T1	5.68 → 0	−5.68	(−5.68, 0.42, 0.48, 0.49, 0.33)	(0, 49.24, 20.91, 10.71, 28.71)	109.57	1.71	0.0
	5.68 → 2	−3.68	(−3.68, 0.27, 0.31, 0.32, 0.21)	(2, 49.09, 20.74, 10.54, 28.59)	110.96	1.11	0.0
	5.68 → 25	19.32	(19.32, −1.41, −1.63, −1.65, −1.10)	(25, 47.41, 18.8, 8.57, 27.28)	127.06	−5.79	0.02
	5.68 → 25.18	19.50	(19.50, −1.43, −1.64, −1.67, −1.11)	(25.18, 47.39, 18.79, 8.55, 27.27)	127.18	−5.85	0.02
T2	48.82 → 0	−48.82	(2.26, −48.82, 9.28, 9.42, 6.28)	(7.94, 0, 29.71, 19.64, 34.66)	91.95	27.23	0.09
	48.82 → 30	−18.82	(0.87, −18.82, 3.58, 3.63, 2.42)	(6.55, 30, 24.01, 13.85, 30.8)	105.21	10.5	0.04
	48.82 → 65	16.18	(−0.75, 16.18, −3.07, −3.12, −2.08)	(4.93, 65, 17.36, 7.1, 26.3)	120.69	−9.02	0.03
	48.82 → 89.79	40.97	(−1.89, 40.97, −7.78, −7.9, −5.27)	(3.78, 89.79, 12.65, 2.32, 23.11)	131.65	−22.84	0.08
T3	20.43 → 0	−20.43	(3.53, 12.59, −20.43, 14.73, 9.82)	(9.21, 61.41, 0, 24.95, 38.2)	133.77	40.67	0.14
	20.43 → 10	−10.43	(1.80, 6.43, −10.43, 7.52, 5.01)	(7.48, 55.25, 10, 17.74, 33.39)	123.86	20.76	0.07
	20.43 → 30	9.57	(−1.65, −5.9, 9.57, −6.9, −4.6)	(4.03, 42.93, 30, 3.32, 23.79)	104.07	−19.04	0.07
	20.43 → 65	44.57	(−5.68, −47.61, 44.57, −10.22, −28.38)	(0, 1.215, 65, 0, 0)	66.22	−91.89	0.53
T4	10.22 → 0	−10.22	(0.75, 2.68, 3.09, −10.22, 2.09)	(6.43, 51.5, 23.52, 0, 30.47)	111.92	8.61	0.03
	10.22 → 3	−7.22	(0.53, 1.89, 2.18, −7.22, 1.48)	(6.21, 50.71, 22.61, 3, 29.86)	112.39	6.08	0.02
	10.22 → 15	4.78	(−0.35, −1.25, −1.44, 4.78, −0.98)	(5.33, 47.57, 18.99, 15, 27.41)	114.3	−4.02	0.01
	10.22 → 105.05	94.83	(−5.68, −34.07, −20.43, 94.83, −26.56)	(0, 14.75, 0, 105.05, 1.82)	121.62	−86.74	0.38
T5	28.38 → 0	−28.38	(2.37, 8.46, 9.75, 9.9, −28.38)	(8.05, 57.28, 30.18, 20.12, 0)	115.63	30.48	0.09
	28.38 → 22	−6.38	(0.53, 1.90, 2.19, 2.26, −6.38)	(6.21, 50.72, 22.62, 12.48, 22)	114.03	6.89	0.02
	28.38 → 32	3.62	(-0.30, −1.08, −1.24, −1.26, 3.62)	(5.38, 47.74, 19.19, 8.96, 32)	113.27	−3.88	0.01
	28.38 → 57	28.62	(-2.39, −8.53, −9.83, −9.98, 28.62)	(3.29, 40.29, 10.6, 0.24, 57)	111.42	−30.72	0.1
	28.38 → 57.9	29.52	(-2.47, −8.82, −10.16, −10.22, 29.52)	(3.21, 40, 10.27, 0,57.9)	111.38	−31.69	0.1
	28.38 → 65	36.62	(−3.31, −11.8, −13.59, −10.22, 36.62)	(2.37, 37.02, 6.84, 0, 65)	111.23	−75.54	0.13
	28.38 → 70	41.62	(−5.68, −1.74, −20.43, −10.22, 41.62)	(0, 47.08, 0, 0, 70)	117.08	−38.07	0.23

**Table 3:** Application of CMAA-LIN model for the selected case mix.

	Revision	δ	Alterations	New Case Mix	ℕ	Impact	Z
T1	5.68 → 0	−5.68	(−5.68, 2.88, 0, 0, 0)	(0, 51.70, 20.43, 10.22, 28.38)	110.73	2.88	0.03
	5.68 → 2	−3.68	(−3.68, 1.88, 0, 0, 0)	(2, 50.69, 20.43, 10.22, 28.38)	111.72	1.88	0.02
	5.68 → 25	19.32	(19.32, 0, −3.84, 0, 0)	(25, 48.82, 16.59, 10.22, 28.38)	129.01	−3.84	0.04
	5.68 → 25.18	19.50	(19.50, 0, −3.88, 0, 0)	(25.18, 48.82, 16.55, 10.22, 28.38)	129.15	−3.88	0.04
T2	48.82 → 0	−48.82	(19.50, −48.82, 0, 0, 22.8)	(25.18, 0, 20.43, 10.22, 51.18)	107.01	42.30	1.10
	48.82 → 30	−18.82	(19.50, −18.82, 0, 0, 5.24)	(25.18, 30, 20.43, 10.22, 33.62)	119.45	24.74	0.85
	48.82 → 65	16.18	(0, 16.18, −6.35, 0, 0)	(5.68, 65, 16.08, 10.22, 28.38)	125.36	−6.35	0.06
	48.82 → 89.79	40.97	(0, 40.97, −16.09, 0, 0)	(5.68, 89.79, 4.34, 10.22, 28.28)	138.31	−16.09	0.16
T3	20.43 → 0	−20.43	(10.5, 31.01, −20.43, 0, 6.51)	(25.18, 79.83, 0, 10.22, 34.89)	150.12	48.02	1.21
	20.43 → 10	−10.43	(19.50, 16.68, −10.43, 0, 0)	(25.18, 65.5, 10, 10.22, 28.38)	139.28	36.19	0.96
	20.43 → 30	9.57	(0, 0, 9.57, −10.22, −5.78)	(5.68, 48.82, 30, 0, 22.6)	107.1	−16	0.18
	20.43 → 65	44.57	(−3.28, −48.82, 44.57, −10.22, −28.38)	(2.4, 0, 65, 0, 0)	67.4	−90.7	1.18
T4	10.22 → 0	−10.22	(19.50, 4.61, 0, −10.22, 0)	(25.18, 53.43, 20.43, 0, 28.38)	127.42	24.12	0.83
	10.22 → 3	−7.22	(19.50, 0.36, 0, −7.22, 0)	(25.18, 49.18, 20.43, 3, 28.38)	126.17	19.87	0.78
	10.22 → 15	4.78	(0, 0, −2.66, 4.78, 0)	(5.68, 48.82, 17.77, 15, 28.38)	115.65	−2.66	0.03
	10.22 → 105.05	94.83	(0, −33.85, −20.43, 94.83, −28.38)	(5.68, 14.96, 0, 105.05, 0)	125.69	−82.66	0.98
T5	28.38 → 0	−28.38	(19.50, 31.01, 0, 5.37, −28.38)	(25.18, 79.83, 20.43, 15.59, 0)	141.03	55.88	1.17
	28.38 → 22	−6.38	(19.50, 1.03, 0, 0, −6.38)	(25.18, 49.85, 20.43, 10.22, 22)	127.68	20.54	0.79
	28.38 → 32	3.62	(0, 0, −2.43, 0, 3.62)	(5.68, 48.82, 18, 10.22, 32)	114.72	−2.43	0.02
	28.38 → 57	28.62	(0, 0, −19.21, 0, 28.62)	(5.68, 48.82, 1.23, 10.22, 57)	122.95	−19.21	0.19
	28.38 → 57.9	29.52	(0, 0, −19.81, 0, 29.52)	(5.68, 48.82, 0.62, 10.22, 57.9)	123.24	−19.81	0.19
	28.38 → 65	36.62	(0, 0, −20.43, −7.45, 36.62)	(5.48, 48.82, 0, 2.78, 65)	122.08	−27.88	0.27
	28.38 → 70	41.62	(−5.68, −1.74, −20.43, −10.22, 41.62)	(0, 47.08, 0, 0, 70)	117.08	−38.07	0.54

**Table 4:** Application of CMAA-SSQ (QP) approach for the selected case mix.

	Revision	δ	Alterations	New Case Mix	ℕ	Impact	Z
T1	5.68 → 25	19.32	(19.32, −0.69, −2.34, −1.34, −0.72)	(25, 48.13, 18.09, 8.89, 27. 66)	100.11	−5.09	0.0
	5.68 → 25.18	19.50	(19.50, −0.7, −2.36, −1.35, −0.73)	(25.18, 48.12, 18.07, 8.86, 27.66)	127.89	−5.14	0.0
T2	48.82 → 65	16.18	(−0.05, 16.18, −4.16, −2.39, −1.28)	(5.63, 65, 16.27, 7.83, 27.10)	121.83	−7.88	0.0
	48.82 → 89.79	40.97	(−0.12, 40.97, −10.53, −6.04, −3.24)	(5.56, 89.79, 9.9, 4.18, 25.14)	134.57	−19.93	0.02
T3	20.43 → 30	9.57	(−0.18, −4.4, 9.57, −8.52, −4.56)	(5.51, 44.43, 30, 1.7, 23.82)	105.46	−17.65	0.01
	20.43 → 65	44.57	(−3.28, −48.82, 44.57, −10.22, −28.38)	(2.4, 0, 65, 0, 0)	67.4	−90.7	0.49
T4	10.22 → 15	4.78	(−0.02, −0.59, −2, 4.78, −0.62)	(5.66, 48.23, 18.42, 15, 27.76)	115.07	−3.234	0
	10.22 → 105.05	94.83	(−1.32, −33.18, −20.43, 94.83, −28.38)	(4.36, 15.64, 0, 105.05, 0)	125.05	−83.31	0.34
T5	28.38 → 32	3.62	(−0.02, −0.5. −1.69, −0.97, 3.62)	(5.66, 48.32, 18.74, 9.25, 32)	113.97	−3.18	0
	28.38 → 57	28.62	(−0.16, −3.95, −13.36, −7.66, 28.62)	(5.23, 44.87, 7.07, 2.56, 57)	116.73	−25.13	0.02
	28.38 → 57.9	29.52	(−0.16, −4.08, −13.78, −7.9, 29.52)	(5.52, 44.74, 6.65, 2.32, 57.9)	117.13	−25.92	0.03
	28.38 → 65	36.62	(−0.2, −5.06, −17.09, −9.81, 36.62)	(5.48, 43.76, 3.34, 0.41, 65)	117.99	−32.16	0.04
	28.38 → 70	41.62	(−5.68, −5.6, −18.91, −10.22, 41.62)	(0, 43.22, 1.52, 0, 70)	114.74	−40.41	0.1

**Table 5:** Application of CMAA-SSQ (SP) approach for the selected case mix.

	Revision	δ	Alterations	New Case Mix	ℕ	Impact	Z
T1	5.68 → 0	−5.68	(−5.68, 2.88, 0, 0, 0)	(0, 51.70, 20.43, 10.22, 28.38)	110.73	2.88	0.0
	5.68 → 2	−3.68	(−3.68, 1.87, 0, 0, 0)	(2, 50.69, 20.43, 10.22, 28.38)	111.72	1.87	0
	5.68 → 25	19.324	(19.32, −0.72, −2.39, −1.26, −0.7)	(25, 48.1, 18.04, 8.96, 27.68)	127.78	−5.07	0.0
	5.68 → 25.18	19.504	(19.50, −0.72, −2.43, −1.26, −0.7)	(25.18, 48.1, 18, 8.96, 27.68)	127.92	−5.11	0.0
T2	48.82 → 0	−48.82	(19.50, −48.82, 0, 0, 22.80)	(25.18, 0, 20.43, 10.22, 51.18)	107.01	42.3	0.71
	48.82 → 30	−18.82	(19.50, −18.82, 0, 0, 5.24)	(25.18, 30, 20.43, 10.22, 33.62)	119.45	24.74	0.61
	48.82 → 65	16.18	(−0.05, 16.18, −4.13, −2.46, −1.26)	(5.63, 65, 16.3, 7.76, 27.12)	121.81	−7.9	0.0
	48.82 → 89.79	40.972	(−0.151, 40.97, −10.52, −6.08, −3.21)	(5.53, 89.79, 9.91, 4.14, 25.17)	134.54	−19.96	0.02
T3	20.43 → 0	−20.43	(19.50, 31.01, −20.43, 0, 6.51)	(25.18, 79.83, 0, 10.22, 34.89)	150.12	57.02	0.73
	20.43 → 10	−10.43	(19.50, 16.68, −10.43, 0, 0)	(25.18, 65.5, 10, 10.22, 28.38)	139.28	36.18	0.63
	20.43 → 30	9.57	(−0.2, −4.48, 9.57, −8.4, −4.61)	(5.48, 44.34, 30, 1.83, 23.77)	105.42	−17.69	0.01
	20.43 → 65	44.57	(−3.28, −48.82, 44.57, −10.22, −28.38)	(2.4, 0, 65, 0, 0)	67.4	−90.7	0.49
T4	10.22 → 0	−10.22	(19.50, 4.61, 0, −10.22, 0)	(25.184, 53.43, 20.43, 0, 28.38)	127.424	24.11	0.6
	10.22 → 3	−7.22	(19.50, 0, 0, −7.22, 0.21)	(25.184, 48.82, 20.43, 3, 28.59)	126.024	19.71	0.6
	10.22 → 15	4.78	(−0.05, −0.54, −2.06, 4.78, −0.56)	(5.63, 48.28, 18.37, 15, 27.82)	115.1	−3.21	0.0
	10.22 → 105.05	94.827	(−1.31, −33.18, −20.43, 94.83, −28.38)	(4.37, 15.64, 0, 105, 0)	125.01	−83.3	0.33
T5	28.38 → 0	−28.38	(19.50, 31.01, 0, 5.37, −28.38)	(25.18, 79.83, 20.43, 15.59, 0)	141.03	55.88	0.72
	28.38 → 22	−6.38	(19.50, 1.03, 0, 0, −6.38)	(25.18, 49.85, 20.43, 10.22, 22)	127.68	20.53	0.6
	28.38 → 32	3.62	(−0.05, −0.54, −1.65, −0.99, 3.62)	(5.63, 48.28, 18.78, 9.23, 32)	113.92	−3.23	0
	28.38 → 57	28.62	(−0.15, −3.94, −13.42, −7.55, 28.62)	(5.53, 44.88, 7.01, 2.67, 57)	117.09	−25.06	0.02
	28.38 → 57.9	29.52	(−0.15, −4.12, −13.73, −7.97, 29.52)	(5.53, 44.7, 6.71, 2.25, 57.9)	117.09	−25.97	0.03
	28.38 → 65	36.62	(−0.2, −5.02, −17.08, −9.86, 36.62)	(5.48, 43.8, 3.35, 0.37, 65)	118	−32.16	0.04
	28.38 → 70	41.62	(−5.68, −5.56, −18.93, −10.22, 41.62)	(0, 43.26, 1.5, 0, 70)	114.76	−40.39	0.1

**Table 6:** Dissimilarity measures for different ϵ vectors.

ϵ	$DISIM_{1}$	$DISIM_{2}$	$DISIM_{3}$	$DISIM_{4}\left(1\right)$	$DISIM_{4}\left(2\right)$
(20, 20, 20, 20, 20)	20%	0.14	0.14	2.33	1.35
(15, 25, 2, 1, 5)	20%	3.32	3.32	7.12	4.56
(6.8, 2, 9.4, 11.8, 5.7)	40%	11.01	10.44	14.66	11.58
(2.5, 9, 6.5, 10.5, 7)	60%	5.18	3.67	8.79	5.21
(2, 2, 2, 2, 2)	80%	19.13	11.83	23.32	13.5
(0.1, 0.1, 0.1, 0.1, 0.1)	100%	461.3	268.28	466.3	270

**Table 7:** New patient type and subtypes.

Sub Type	Mix (%)	Summary	Pathway	Length of Stay
T6–1 (mild)	45	Patients with mild upper respiratory tract infection	Ward	0.25 days in a ward
T6–2 (moderate)	35	Patients requiring hospitalization, with pneumonia and with/without the need for oxygen	Ward	5 days in a ward
T6–3 (severe)	15	Patients who need ICU treatment and require non-invasive or invasive mechanical ventilation, or with acute respiratory distress and/or non-pulmonary involvement	(Ward, ICU, Ward)	2 days in ward prior to ICU, 5 days in ICU + 7 days in a ward
T6–4 (critical)	5	Patients who need immunomodulatory therapy or with multi-organ failure and/or cytokine storm	(ICU, Ward)	14 days in ICU + 7 days in a ward

**Table 8:** Revised hospital layout.

Ward	Repurposed for Covid	#Beds	Used For	Ward	Repurposed for COVID	#Beds	Used For
W1	✓	2	T6	W4	✕	14	T4–1, T5–1, T1–1, T1–2, T2–1
W2	✕	5	T1–1, T1–2, T2–1	W5	✓	3	T6
W3	✕	10	T3–1, T3–2, T3–3	W6	✓	6	T6

**Table 9:** New patient type information and bounds.

Type	${\bar{n}}^{1}$		# Subtypes	Subtype	Subtype Mix (%)	(ic, sur, postop) Time (# h)	Wards Used
	(orig)	(new)					
1	201.47	1000	2	1–1	70	(0, 1.2, 17.86)	W2, W4
				1–2	30	(6, 1.25,8.35)	W2, W4
2	718.33	1000	1	2–1	100	(0, 2.4, 16.31)	W2, W4
3	523.82	523.82	3	3–1	25	(0, 6.5, 12.94)	W3
				3–2	40	(0, 4.56, 12.39)	W3
				3–3	35	(0, 7.6, 5.54)	W3
4	840.38	840.38	1	4–1	100	(0, 3.4, 18.99)	W4
5	560	560	1	5–1	100	(12, 4.1, 22.81)	W4
6	na	172.91	4	6–1	45	(0, 0, 6)	W1, W5, W6
				6–2	35	(0, 0, 120)	W1, W5, W6
				6–3	15	(120, 0, 216)	W1, W5, W6
				6–4	5	(336, 0, 168)	W1, W5, W6

**Table 10:** Application of CMAA-EQ approach.

δ	Alterations	$\mathrm{New}\;\mathrm{Case}\;\mathrm{Mix}\;(n^{1}$) ℕ (ℕ−δ)	Ward Util (%) (ICU, OT, W1, W2, W3, W4, W5, W6)
50	(−0.0025, −0.0026, −0.001, −0.002, −0.001, 100)	(45.41, 390.54, 163.48, 81.74, 227.06, 50) 958.22 (908.22)	(67.66, 100, 0, 9.02, 19.75, 81.73, 0, 53.01)
100	(−0.0025, −0.0026, −0.001, −0.002, −0.001, 100)	(45.41, 390.53, 163.48, 81.74, 227.06, 100) 1008.2 (908.22)	(93.55, 100, 100, 0, 19.75, 84.95, 100, 22.69)
125	(−45.41, −52.79, −27.65, −44.36, −29.56, 125)	(0, 337.75, 135.83, 37.38, 197.5, 125) 833.46 (708.46)	(100, 80.54, 100, 0, 16.41, 66.28, 100, 49.2)
150	(−45.41, −182.25, −95.47, −81.74, −102.06, 150)	(0, 208.29, 68.01, 0, 125, 150) 551.3 (401.3)	(100, 44.62, 100, 0, 8.22, 35.59, 100, 75.71)
172	(−45.41, −296.18, −155.14, −81.74, −165.86, 172)	(0, 94.36, 8.34, 0, 61.2, 172) 335.9 (163.9)	(100, 16.51, 100, 0, 1, 18.14, 100, 99.03)

**Table 11:** Application of CMAA-LIN approach.

δ	Alterations	$\mathrm{New}\;\mathrm{Case}\;\mathrm{Mix}\;(n^{1}$) ℕ (ℕ−δ)	Ward Util (%) (ICU, OT, W1, W2, W3, W4, W5, W6)
50	(0, 0, −0.005, 0, 0, 50)	(45.41, 390.54, 163.48, 81.74, 227.06, 50) 958.22 (908.22)	(67.66, 100, 0, 9.02, 19.75, 81.73, 0, 53.01)
100	(0, 0, −0.005, 0, 0, 100)	(45.41, 390.53, 163.48, 81.74, 227.06, 100) 1008.2 (908.22)	(93.55, 100, 100, 0, 19.75, 84.95, 44.2, 50.6)
125	(0, 0, 0, 0, −36.37, 125)	(45.41, 390.54, 163.48, 81.74, 190.69, 125) 996.86 (871.86)	(100, 95.34, 100, 0, 19.75, 79.75, 89.58, 54.41)
150	(0, 0, 0, 0, −108.87, 150)	(45.41, 390.54, 163.48, 81.74, 118.19, 150) 949.36 (799.36)	(100, 86.05, 100, 0, 19.75, 69.38, 100, 75.71)
172	(0, 0, 0, 0, −172.67, 172)	(45.41, 390.54, 163.48, 81.74, 54.39, 172) 907.56 (735.56)	(100, 77.88, 100, 0, 19.75, 60.25, 100, 99.03)

**Table 12:** Application of CMAA-SSQ(QP) approach.

δ	Alterations	$\mathrm{New}\;\mathrm{Case}\;\mathrm{Mix}\;(n^{1}$) ℕ (ℕ−δ)	Ward Util (%) (ICU, OT, W1, W2, W3, W4, W5, W6)
50	(−0.01, −0.01, −0.01, −0.01, −0.01, 50)	(45.41, 390.54, 163.48, 81.74, 227.06, 50) 958.17 (908.17)	(67.66, 100, 0, 0, 19.75, 84.94, 0, 53.01)
100	(−0.01, −0.01, −0.01, −0.01, −0.01, 100)	(45.41, 390.53, 163.48, 81.74, 227.06, 100) 1008.2 (908.22)	(93.55, 100, 66.77, 0, 19.75, 84.95, 62.67, 52.43)
125	(−16.23, −0.012, −0.016, −0.014, −33.94, 125)	(29.18, 390.52, 163.46, 81.73, 193.12, 125) 983.01 (858.01)	(100, 95.03, 72.98, 0, 19.75, 78.69, 71.91, 72.25)
150	(−45.41, −0.012, −0.011, −0.011, −102.06, 150)	(0, 390.53, 163.47, 81.73, 125, 150) 910.73 (760.7)	(100, 85.2, 82.15, 0, 19.75, 66.44, 84.5, 89.41)
172	(−41.41, −0.014, −0.012, −0.013, −165.86, 172)	(0, 390.53, 163.47, 81.73, 61.12, 172) 868.92 (696.92)	(100, 77.02, 98.81, 0, 19.75, 57.31, 57.31, 99.29, 9.79)

## Data Availability

All data is present in this paper.

## Appendix A

Case Mix Planning (Capacity) Model

$n_{g}^{1}$: Flow of patients of type g

$n_{g,p}^{2}$: Flow of patients of subtype (g,p)

$\beta_{a,i}$: Flow of activity a at location i

(A1) $$\mathrm{Maximize}\;\mathbb{N}=\sum_{g}n_{g}^{1}\;(\mathrm{Total}\mathrm{flow})$$

Subject To:

(A2) $$n_{g}^{1}=\sum_{p\in P_{g}}\;\left(n_{g,p}^{2}\right)\;\forall g\in G(\mathrm{Aggregated}\mathrm{flow})$$

(A3) $$n_{g,p}^{2}=\sum_{i\in I_{a}}\beta_{a,i}\;\forall g\in G,p\in P_{g},a\in A_{g,p}\;(\mathrm{Allocations})$$

(A4) $$U_{i}=\sum_{a\in A_{i}}\beta_{a,i}t_{a}\leq T_{i}\;\forall i\in I\;(\mathrm{Usage}\mathrm{and}\mathrm{Time}\mathrm{Availability})$$

(A5) $$n_{g}^{1}\geq 0;\;n_{g,p}^{2}\geq 0;\;\;\forall g\in G,p\in P_{g}\;(\mathrm{Positive}\mathrm{flows})$$

(A6) $$\beta_{a,i}\geq 0\;\forall a\in A,i\in I\;(\mathrm{Positive}\mathrm{allocations})$$

(A7) $$n_{g,p}^{2}\geq \mu_{g,p}^{2}n_{g}^{1}\;\forall g\in G,p\in P_{g}\;(\mathrm{Sub}\mathrm{mix}\mathrm{adherence})$$

(A8) $$n_{g}^{1}\geq \mu_{g}^{1}\mathbb{N}\;\forall g\in G\;(\mathrm{Case}\;\mathrm{mix}\;\mathrm{adherence})\;$$

Constraints (A1)–(A3) provide basic bookkeeping. These equations model the inherent relationship between the flow of patients of each type and subtype, and the allocation of resources. Resource usage is restricted by constraint (A4). Constraints (A5) and (A6) enforce positive flows. Finally, case mix and sub mix are enforced by (A7) and (A8).
